# Multi-Parameter Vital Sign Telemedicine System Using Web Socket for COVID-19 Pandemics

**DOI:** 10.3390/healthcare9030285

**Published:** 2021-03-05

**Authors:** Chuchart Pintavirooj, Tanapon Keatsamarn, Treesukon Treebupachatsakul

**Affiliations:** School of Engineering, King Mongkut Institute of Technology Ladkrabang, Bangkok 10520, Thailand; 60601222@kmitl.ac.th (T.K.); treesukon.tr@kmitl.ac.th (T.T.)

**Keywords:** cloud platform, ECG, plethysmogram, vital sign, patient monitor, NIBP

## Abstract

Telemedicine has become an increasingly important part of the modern healthcare infrastructure, especially in the present situation with the COVID-19 pandemics. Many cloud platforms have been used intensively for Telemedicine. The most popular ones include PubNub, Amazon Web Service, Google Cloud Platform and Microsoft Azure. One of the crucial challenges of telemedicine is the real-time application monitoring for the vital sign. The commercial platform is, by far, not suitable for real-time applications. The alternative is to design a web-based application exploiting Web Socket. This research paper concerns the real-time six-parameter vital-sign monitoring using a web-based application. The six vital-sign parameters are electrocardiogram, temperature, plethysmogram, percent saturation oxygen, blood pressure and heart rate. The six vital-sign parameters were encoded in a web server site and sent to a client site upon logging on. The encoded parameters were then decoded into six vital sign signals. Our proposed multi-parameter vital-sign telemedicine system using Web Socket has successfully remotely monitored the six-parameter vital signs on 4G mobile network with a latency of less than 5 milliseconds.

## 1. Introduction

The new COVID-19 disease was first identified in China, at the end of 2019, and has been spread rapidly all over the world. It has been projected that, by March 2021, the number of infections could reach 300 million cases and more than two million deaths [1]. To prevent the rapid spread of COVID-19, many countries declare curfews, quarantine and restrictions. It has been estimated that half of the world population will be under partial lockdowns or lockdowns by mid-2021 [1]. As a number of COVID-19 infective cases become significantly increased, the hospital facilities are running at full capacity, with 80% serving COVID-19 related diseases. A patient with mild COVID-19 symptom or patient with other diseases are then forced to be treated at home. During the pandemics of COVID-19, telemedicine has been playing an important role in healthcare technology, especially for bed-ridden patients, patients with mild COVID-19 symptoms or patients with other diseases.

Telemedicine refers to a service of caring for patient remotely via a real-time bi-direction communication between a healthcare provider and patient, using telecommunications technology [2,3,4,5,6,7]. With telemedicine, patients can consult, receive treatment, diagnosis and evaluation comfortably, at home, without being physically present with each other. Telemedicine can be roughly classified based on two principles: (i) types of interaction between healthcare provider and patient and (ii) types of communicated information. Types of interactions can be further classified into off-line communication and real-time communication. For off-line communication, the information will be acquired and recorded before being sent for interpretation. The information sent by off-line communication is not time-sensitive. Digital radiograph [8], email, electrocardiogram (ECG) and recorded on paper are examples of off-line communication. Real-time communication, by contrast, is used when the information is time-sensitive data. There will be no noticeable delay between the time when the information is collected and sent in patient (provider) site and when the information is being displayed on the provider (patient) site. Tele ECG [9] is an example of real-time communication. Real-time communication can also further classify based on a rate of acquisition. For a low acquisition rate, a commercial cloud platform, including PubNub, Amazon Web Service, Google Cloud Platform and Microsoft Azure, can be applied. The latency is defined as the delay time between end-to-end transmission. For PubNub, for example, latency of 30–40 ms can be achieved, and hence the maximum frequency content that can be sent without aliasing a problem with PubNub is only 12.5 Hz [10]. For high-acquisition-rate real-time data communication, such as electrocardiogram (ECG), commercial cloud platform is not suitable, as the recommended sampling frequency for ECG is 1000 Hz. In such cases, the latency should not exceed 1 ms. Another criterion used for telemedicine classification is based on types of communicated information, i.e., the dimension of information. One-dimension information included, for example, the ECG signal and the audio signal, where two-dimensional information is the image such as the radiographic image. The two-dimensional data may also refer to the time sequence of one-dimensional data. The three-dimensional data may refer to the video pictures, which are the motion of two-dimensional data.

Real-time telemedicine associated ECG signal is a challenging problem that captures researcher attention over the past decades. Carlos and Oliveira [11] exploited Internet Message Access Protocol (IMAP), Post Office Protocol (POP) and Simple Mail Transfer Protocol (SMTP) of electronic mailbox, to develop a plug-and-play telemedicine cardiologic application supporting the core of the telemedicine information system associated with ECG and medical images. A security model was also developed, to ensure data privacy and confidentiality. The proposed off-line ECG telemedicine provided a secure telemedicine platform that is easily installed by end-users. The proposed system of Carlos and Oliveira is limited only the printout of ECG data. Sukanesh et al. [12] developed a mobile, basic and well-known technique to detect abnormalities in ECG and send alert to inform doctor and patient in advance. Even though the Sukanesh et al. proposed module is a real-time ECG monitoring system, but only the alert Short Message Service (SMS) message is sent to the doctor through the Global System for Mobile (GSM). Fensli et al. [13] developed a wearable tele-electrocardiogram to send a digitized one-leaded two-wired ECG sampled at 500 Hz, using 10-bit analog to digital converter (ADC) to a hospital via Hand-Held Device (HHD). The system is used for arrhythmia detection. In the presence of arrhythmia, a one-minute stored ECG datum in HHD is transmitted to a doctor via General Packet Radio Service (GPRS) communication for further investigation. The drawback of proposed technique of Fensli et al. is that the system can only send the recorded ECG not the real-time ECG. Jatmiko et al. [14] developed a tele-ECG system on a web server, using java platform and Java EE framework to monitor heart diseases, and sent an alert to the doctor at the onset of heart failure. Implemented on the field programmable gate array (FPGA) board, the system consists of ECG acquisition, feature extraction, data compression, classification algorithm and networking hardware. Similar to a work of Sukanesh et al. [12], the system of Jatmiko et al. can send only real-time alert message to appropriate practitioner to quickly respond to patient. Sufi and Khalil [15] proposed a secured ECG transmission for real-time telemonitoring by acquiring sampled ECG data, encoding ECG data and sending the encoded ECG data to the hospital. The encoding scheme of Sufi and Khalil not only provided high-security transmission but also proposed a higher compression ratio for data transmission of up to 20.06, making it feasible for real-time telemonitoring. Hinestroza et al. [16] developed an ECG acquisition board that can communicate with android phones by using Bluetooth communication. The received ECG data on the internet-connected mobile phone are then sent to a healthcare provider, for further diagnosis. Even though the system of Sufi and Khalil [15] and Hinestroza et al. [16] can successfully send the real-time ECG from patient to caretaker, but only one channel of ECG can be sent. Zhang et al. [17] proposed a remote mobile health-monitoring system based on the smart phone and browser/server structure. The system can remotely monitor patient physiologic data, including respiration rate and heart rate, patient location and patient status, on the mobile smart phone. Naik et al. [18] extended the research work that was done by Zhang et al. [17] by including more physiological data, such as temperature, percent saturation oxygen (SpO2), ECG, blood pressure and etCO2. The mobile health monitoring system purposed by Naik et al. and Zhang et al. operated on the android platform of the smart phone, where resources are limited. The extension for multiple users and the improved performance of the system are, hence, not feasible.

This paper concentrates on a real-time telemedicine, to monitor vital-sign signals. The salient aspects and/or contributions of this paper are enumerated as follows.

(1) This paper presents a development of a real-time, low-cost, multi-parameter and easy-to-implement vital-sign telemedicine which is very suitable for the current situation of the COVID-19 pandemic.

(2) A novelty of our research is to use is LINUX Web Socket package for real-time vital-sign telemedicine, to communicate the data between the server and the client. To our best knowledge, although Web Socket has been used for real-time application [19,20,21], never has it been used for vital-sign telemonitoring.

(3) What differentiates our research from previous work is that our proposed real-time telemedicine is capable of monitoring six-parameter vital signals simultaneously, i.e., ECG, plethysmogram, SpO2, temperature, blood pressure and heart rate.

The rest of the paper is organized as follows. Section 2, Section 3, Section 4 and Section 5 describe the acquisition system of ECG, plethysmogram, body temperature and blood pressure, respectively. Vital-signal signal conditioning with digital filtering is explained in Section 6. Section 7 explains the telemedicine system including web server and web socket. Section 8 concerns the performance test of the proposed vital-sign telemedicine system. Discussion and conclusion are in Section 9 and Section 10, respectively.

## 2. ECG Data Acquisition and Signal Conditioning

**Figure 1:**
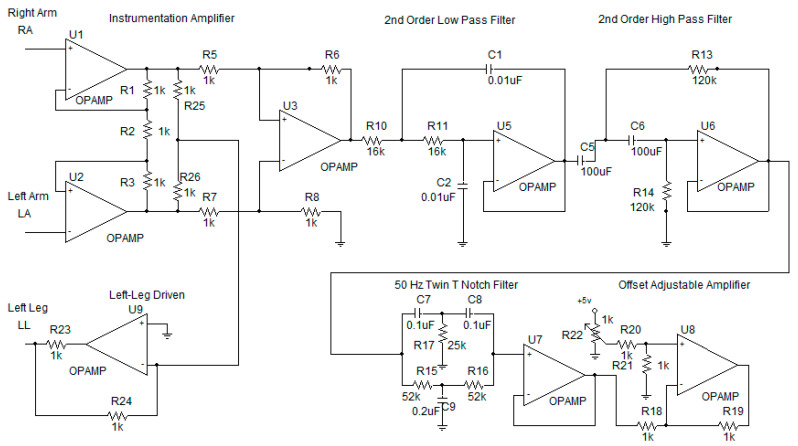
Electrocardiogram (ECG) acquisition circuit.

The ECG acquisition and signal-conditioning circuit are shown in Figure 1, consisting of an (i) instrumentation amplifier, (ii) right-leg driven, (iii) low-pass filter and high-pass filter, (iv) 50 Hz notch filter and (v) offset-adjustable amplifier. Details are as follows.

(i) Instrumentation Amplifier [22]

Commonly known as a biomedical amplifier, the instrumentation amplifier consists on OpAmps U1, U2 and U3. The circuit is immune to noise and hence is widely used to amplify biological signals where the magnitude is very small. To measure how the circuit is immune to noise, common-mode rejection ratio (ratio of differential mode and common-mode gain) is used. With the perfect balance of upper-half resistor (R1, R5 and R6) and lower-half resistor (R3, R7 and R8), the instrumentation amplifier can achieve a Common Mode Rejection Ratio (CMRR) [22] of 75 dB. The gain of instrumentation amplifier can be computed by the following:

(1) $$G=\left(1+\frac{2R1}{R2}\right)\frac{R6}{R5}$$

We use a gain of 25, which can effectively amplify 0.5–3 mV ECG signals from patient’s body.

(ii) Right-Leg Driven [22]

Right-leg driven (DRL) depicted in circuit as U9 is implemented to reduce interference due to common-mode voltage and to provide patient safety. In general, to improve CMRR, the common mode gain caused by the interference has to be reduced. Normally, the interference can be caused by the operation of inductance load equipment and high-power line nearby. To reduce the common mode gain, i.e., increase CMRR, the common mode signal is trapped between R25 and R26 and driven back to the body.

(iii) Low-Pass Filter and High-Pass Filter [22]

Spectrum of ECG is between 0.01 and 100 Hz [22]. Any signal beyond this range will be considered as a noise. Both a low-pass filter and high-pass filter are used to make sure that the signal being amplifier is only the ECG. The 2nd-order low-pass filter and 2nd-order high-pass filter are depicted as OpAmps U5 and U6, respectively. The cutoff frequency of high-pass filter and low-pass filter is set as 0.01 Hz and 100 Hz, respectively. The low-pass filter cut-off frequency can be computed by the following:

(2) $$fc=\frac{1}{2\pi \sqrt{C1C2R10R11}}$$

where that of the high-pass-filter cutoff frequency can be computed by the following:

(3) $$fc=\frac{1}{2\pi \sqrt{C5C6R13R14}}$$

(iv) 50 Hz Notch Filter [22]

Even though the low-pass filter and high-pass filter are used to ensure that only the ECG signal of which the frequency is between 0.01 and 100 Hz is amplified, unfortunately the 50 Hz noise, which is mainly caused by line-voltage interference, can pass into the circuit. To remove the 50 Hz noise, a twin-t notch filter is used. This is shown as C7, C8, C9, R15, R16, R17 and U7 in the ECG circuit

(v) Offset-Adjustable Amplifier [22]

The final stage of ECG signal is offset-adjustable amplifier U8. Its function is to finally adjust the gain and the offset voltage to the suitable level, before applying to the microcontroller.

To provide better performance, high precision, high stability and low power consumption, the OpAmps used in the ECG acquisition circuit will be military-grade OpAmps, where the resistor will be one percent error resistor and capacitor will be tantalum capacitor.

## 3. Plethysmogram Data Acquisition

MAX30100 is a pulse oximetry and heartrate module that is designed for wearable devices. It consists of an 880 nm infrared light emission diode (IR LED), 660 nm red light emission diode Red (LED) and photodetector and signal conditional circuit, including (Direct Current (DC) optimized optics, DC Ambient Light Rejection and low-noise analog-signal processing. MAX30100 is widely used in a minimum-power-consumption wearable device that can provide a high-accuracy measurement of peripheral oxygen saturation (SpO2) and heart rate (HR). To optimize performance for various site of measurement, LED current and LED pulse can be adjusted. The adjustable range of LED current and LED pulse is 0 mA to 50 mA and 200 µs to 1.6 ms, respectively.

However, MAX30100 provides all the essential components, the output coming from MAX30100 is still needed to be accustomed. The output signal must be modified through these following libraries.

The first library concerns DC removal. To remove the DC component, signal averaging is estimated at some instance of time and later subtracted from the original signal. DC removal can be done by applying infinite impulse response filter (IIR).

The second library is to balance IR and Red currents and is used to control the intensity of IR and RED. A mechanism of this library is to first check the difference between I_DCred_ and I_DCIR_ reading. After that, if the value of I_RED_ is greater than I_IR_ then it will decrease the I_RED_ current but if the I_RED_ value is less than the IIR then it will increase the I_RED_ current.

The third library is the variable referred to the difference between the electrical current level supply of red and IR light emitter or I_DCred_ and I_DCIR_ which is called MAGIC_ACCEPTABLE_INTENSITY_DIFF (MAID). The suitable value of the MAID is important because if the MAID is too high, the oxygen saturation level will be oversaturated. However, on the other hand, if the MAID is too low, oscillation will occur. Lastly, when the MAID is suitable, the IDC_red_ and IDC_IR_ will balance out and stay stable.

The fourth library is the low-pass filter (LPF) or the so-called mean filter. The aim of LPF is used to smoothen the signal and hence improve the ability to detect the pulse of the signal.

The last library is the Butterworth band pass filter (BPF), which allows only the signal in the desired frequency to pass through and remove the high-frequency signal, especially the higher-level harmonies of the signal. BPF also improves the performance of pulse detection by filtering the IR signal.

After the signal goes through the previous libraries, it is ready to be used by other libraries, to find several biological values, 

Beat detection is the library that is used to determine human heart rate. To determine heartrate, signal thresholding is applied. When the signal reaches the setting threshold, starting beat timestamp is marked. The signal is followed until the signal reaches threshold again. The new timestamp is then marked as ending beat timestamp. BPM is then calculated as follows: BPM = 60,000 ms/(ending beat timestamp—starting beat timestamp).

Ratio I library is used to estimate ratio R according to the principle of a pulse oximeter, which is defined as *R = log (IACred) λ1/log(IACIR)∙λ2,* where *IACred* is the root mean squared red signal. λ1 is the RED wavelength at 660 nm. *IACIR* is the root mean squared IR signal. λ 2 is the IIR wavelength at 880 nm.

Oxygen Saturation (SpO2), a standard model of computing SpO2 is as follows: SpO2 = 110.0 − 25 * R.

## 4. Human Body Temperature Acquisition

**Figure 2:**
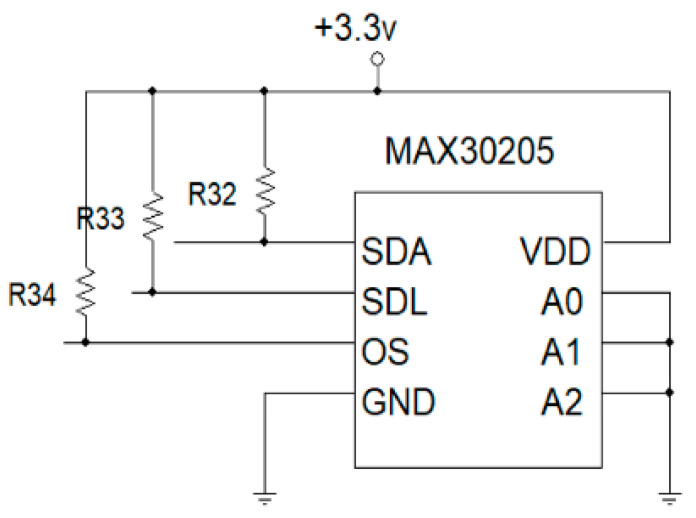
Temperature-sensor wiring diagram.

Body temperature sensor is designed by using a high-accuracy, high-resolution MAX30205 temperature module. The device consists of three main components: a temperature sensor, analog-to-digital converter (ADC) and I2C communication. The temperature sensor converts temperature to electrical signal. The converted signal is then digitized, using a 16-bit sigma-delta analog-to-digital converter (ADC). A 2-wire I2C communication is used to read temperature output and write configuration data to the device. The sensor power consumption is pretty low, using a 2.7 to 3.3 V supply voltage range and low 600 µA supply current. Due to the low supply current, the effects of device self-heating on temperature errors are minimal. A lockup-protected I2C-compatible interface makes MAX30205 ideal for wearable fitness and medical applications. MAX30205 temperature sensor wiring diagram is shown in Figure 2. The SDA and SDL pin will connect to microcontroller via I2C.

## 5. Blood Pressure Estimation

Conventional noninvasive blood pressure (NIBP) measurement uses inflatable cuff wrapping around patient arm by pumping air into the cuff above the systolic pressure and later releasing the pressure. The oscillometric pulse detected with pressure sensor sensing air in the inflatable cuff can then be used to estimate systolic, diastolic and average blood pressure. Inflatable cuff, however, is not feasible for real-time monitoring for blood pressure, due to many reasons. First, high pressure in the cuff could cause pain around the patient’s arm, especially when the blood-pressure acquisition interval is short. Second, an inflatable cuff with attached tubes obscures the national motion of patient body. Third, the long blood pressure acquisition time could not send hypertension notification to the caretaker on time. We hence used the concept of pulse transit time to acquire arterial blood pressure in real time [23,24,25,26,27].

**Figure 3:**
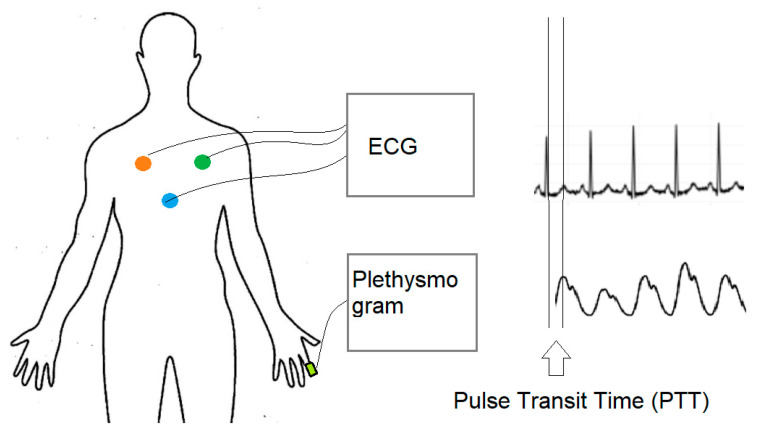
Pulse transit time (PTT).

Pulse transit time (PTT), as shown in Figure 3, is the time delay between the R-peak of ECG and the peak of plethysmogram wave. The onset of the R-peak is the time when the heart pumps blood from the left ventricle to extremities, where the onset of plethysmogram peak determines the time arterial blood arriving at the fingertip. Many factors affect pulse transit time, including vessel length, blood density, inner radius of the vessel, vessel wall thickness and elastic modulus of vascular wall [24]. Most factors are constant parameters, except for blood pressure. There exist many models to establish the relation between blood pressure and pulse transit time, including linear model, logarithm model, inverse model and inverse square model [27], as shown in Equations (4)–(7), respectively.

Linear Model:

*BP* = *a PTT* + *b* (4)

Logarithm Model:

*BP* = *a ln(PTT)* + *b* (5)

Inverse Square Model:

(6) $$BP=\frac{a}{PTT^{2}}+b$$

Inverse Model:

(7) $$BP=\frac{a}{PTT}+b$$

where *BP* is blood pressure, *PTT* is pulse transit time, and *a* and *b* are coefficients to be estimated.

In our research, we opted to use the logarithm model, due to the minimum-error estimation [28]. In the model, the relation between blood pressure (*BP*) and pulse transit time (PTT) can be written as follows [24]:

(8) $$BP=-\frac{2}{\propto }lnPTT+\frac{ln\frac{2r\rho L^{2}}{hE0}}{\alpha }$$

where *L* is the vessel length, *PTT* is the time that a pressure pulse spends in transmitting through that length, *ρ* is the blood density, *r* is the inner radius of the vessel, *h* is the vessel wall thickness, α is constant term related of elasticity and E_0_ is the elastic modulus of vascular wall. Constant parameters in Equation (8) are unique for each individual and can be estimated by fitting to the known *n* dataset of blood pressure and *PTT*. Rewrite Equation (8) in matrix form, as follows:

*Y* = *XA* (9)

where *Y* is the vector of *n* known value of blood pressures, defined as follows:

$$\left[\begin{array}{c} BP1\\ BP2\\ \begin{array}{c} BP3\\ \vdots \\ BPn \end{array} \end{array}\right]$$

*X* is the vector of *n* corresponding known value of PTT, defined as follows:

$$\left[\begin{array}{cc} lnPTT1 & 1\\ \begin{array}{c} lnPTT2\\ \vdots \\ lnPTTn \end{array} & \begin{array}{c} 1\\ \vdots \\ 1 \end{array} \end{array}\right]$$

Moreover, *A* is the vector of constant terms, defined as follows:

$$\left[\begin{array}{c} -\frac{2}{\alpha }\\ \frac{ln\frac{2r\rho L^{2}}{hE0}}{\alpha } \end{array}\right]$$

The constant-parameter vector *A* can then be estimated by using the minimized mean square error [24], as follows:

*A* = (*X^T^X*)^−1^(*X^T^Y*) (10)

Using the estimated constant term derived from Equation (10), blood pressure can then be estimated by using Equation (8). It has been shown that *PTT* can be used to estimated blood pressure, with an error less than 2–3 mmHg. [26]

## 6. Vital-Signal Signal Conditioning with Digital Filtering

**Figure 4:**
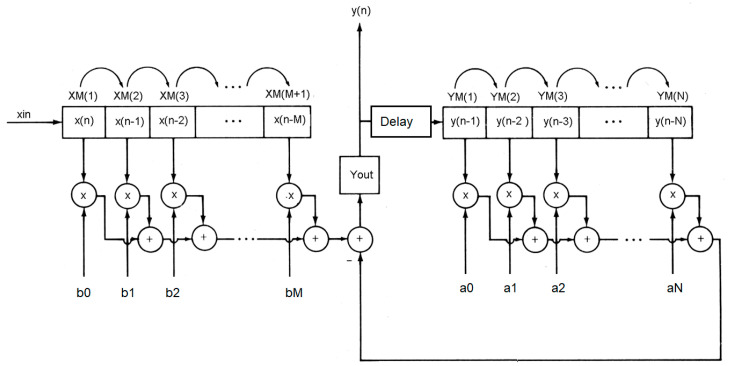
Digital filtering system [30].

Even though vital-sign analog signal conditioning circuits explained in Section 2 can significantly remove the noise inference before transmitting the signal. The received signal at the server site may be attacked by noise, especially 50 Hz line voltage interference. We hence improved signal conditioning further, by using digital filtering [29]. In general, the digital filtering system can be described by the difference equation, as follows:

(11) $$y\left(n\right)=-\overset{N}{\underset{k=1}{\sum }}a_{k}y\left(n-k\right)+\overset{M}{\underset{k=0}{\sum }}b_{k}x\left(n-k\right)$$

where *x(n)* is digitized input signal, *y(n)* is digitized output, and *a_k_* and b_k_ are the digital filtering coefficients of order *N* and *M*, respectively. The digital filtering system in Equation (11) can be diagramed as in Figure 4. In the figure, the new digital input data are inserted in the shifted signal data array. The shifted input data are then multiplied with the filter coefficient *b_k_* and then minus the previous computed output. The current computed output after delaying one-time unit will be shifted into output digital array before multiplying with filter coefficient *a_k_*_,_ to derive the previous output. To implement digital filtering, the following pseudo computer code in Algorithms 1 is employed, assuming that the order of input and output coefficient is equal and set to *M*. In our system, digital filtering is performed at the server Node.js site.
**Algorithms 1:** Digital Filtering     xm(0) = xin
     sum = 0.0
     for k = M:−1:1
        sum = sum + b(k)* xm(k)
        sum = sum + a(k)*ym(k)
        xm(k) = xm(k-1)
        ym(k) = xm(k-1)
     end
     yout = sum + b(0)*xm(0)
     ym(1) = yout

**Figure 5:**
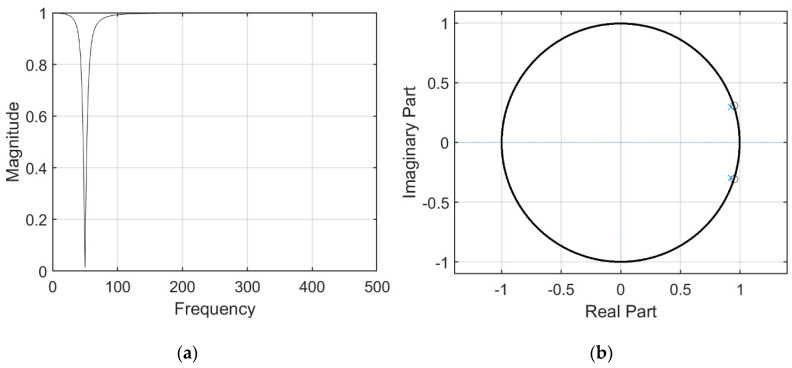
Third-order 50 Hz notch filter with ak = [1.0000–1.8451 0.9391], bk = [0.9695–1.8451 0.9695] and sampling frequency = 1000 Hz: (**a**) frequency response and (**b**) pole-zero pattern plot.

We used MATLAB to design a digital 50 Hz notch filter of order 3. The frequency response and pole-zero pattern plot are shown in Figure 5.

## 7. Vital-Sign Telemedicine System

**Figure 6:**
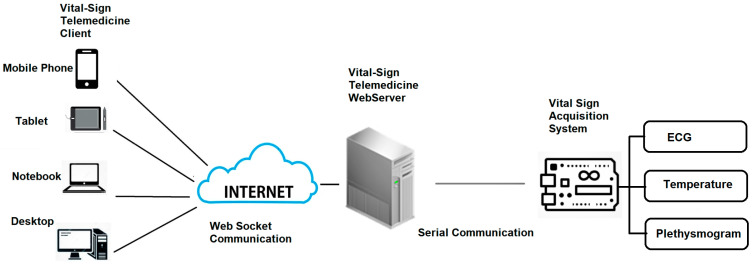
Vital-sign telemedicine system.

The vital-sign telemedicine system depicted in Figure 6 consists of three main parts: (i) vital-sign acquisition system, (ii) vital-sign telemedicine web server and (iii) vital-sign telemedicine web client.

(i) Vital-Sign Acquisition System

The function of vital-sign telemedicine acquisition system is to acquire vital signs that will be used for monitoring. The vital-sign telemedicine acquisition system is subdivided into four main parts: (i) vital-sign microcontroller, (ii) ECG acquisition board, (iii) plethysmogram acquisition board and (iv) body-temperature acquisition board.

For the vital-sign microcontroller, we opted to use Arduino Mega 2560, which is a microcontroller board that has 54 digital input/output pins (of which 14 can be used as Pulse-with modulation (PWM) outputs), 16 analog inputs, four serial communication ports, a 16-megahertz (MHz) crystal oscillator, two I2C communication and 256 kilobyte (KB) flash memory. The plethysmogram acquisition board and body-temperature acquisition board are communicated with vital-sign microcontroller via I2C port. The ECG acquisition port is connected to vital-sign microcontroller via analog inputs. The vital-sign microcontroller sends the information for the six vital signs, i.e., ECG, plethysmogram, percent saturation oxygen, heart rate, blood pressure and body temperature, to the vital-sign web server, using serial communication.

**Figure 7:**
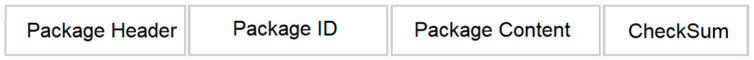
Communication package.

To communicate the data from the vital-sign telemedicine acquisition system to vital-sign telemedicine web server, and finally to the vital-sign telemedicine web client, we designed a communication package, which is shown in Figure 7.

**Figure 8:**
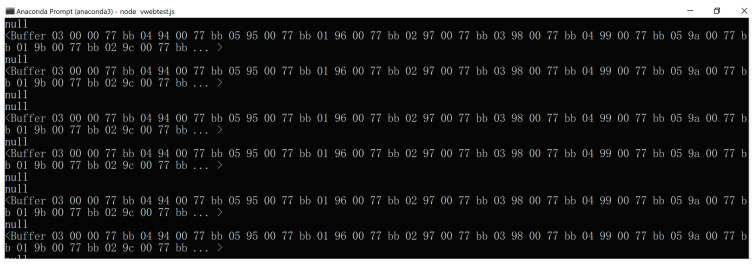
Broadcasting data on Node.js vital-sign web server.

The package header contains two bytes, which are 0 X77 0Xbb. The package ID integrates the vital-sign data type. The package ID from 0 × 00 to 0 × 05 is for ECG, heart rate, plethysmogram, SpO2, blood pressure and body temperature, respectively. The package content is for 1-byte of real data. Checksum is for validation. Checksum is the sum of data from package ID to package content. One dataset for six parameters (ECG, heart rate, plethysmogram, SpO2, blood pressure and body temperature) requires 30 bytes (5 bytes × six vital-sign parameters). A sample of the broadcasting data is shown in Figure 8. To broadcast data, the package is sent to the client’s browser when the client has logged in to the vital-sign web server.

(ii) Vital-Sign Web Server

Located at the patient’s home, the vital-sign web server is operated in the Linux platform, using Node.js. One patient server will have one URL. If the caretaker would like to access the patient’s server, he/she can open the internet browser to access the specific URL. Once logged in, the caretaker can access the patient’s monitor page. To monitor multiple patients, multiple internet browser can be opened simultaneously. There are two main functions of the web server. First, it communicates with the vital-sign telemedicine acquisition system via serial communication. Second, it waits for the client to log in. Once the client has logged in, it will broadcast the received data to the client via index html file. The process of vital-sign web server using Web Socket can be summarized as follows.

♦ Step (i): Start serial communication protocol, to establish serial communication with vital-sign telemedicine acquisition system.
♦ Step (ii): Start Web Socket package to prepare for client connection via the setting port.
♦ Step (iii): Wait for the client to connect.
♦ Step (iv): If the client connects, broadcast the received data and start the index.html file.

The Node.js server code and explanation are shown in Appendix A.

(iii) Vital-sign Web Client

The vital-sign web client will initiate the index.html file. The vital-sign data are sent from the sever to the client via Web Socket. Once received, the vital-sign data are decoded and plotted on the html canvas. The process of vital-sign web client, using Web Socket, can be summarized as follows.

- Step (i): Prepare the call-back function to run if there are data sent from the server by the Web Socket package.
- Step (ii): If data are received, encode the data into separated parameters.

Plot the new ECG data and Plethysmogram data on the prepared html canvas.

Update the displayed parameters for heart rate, SpO2, blood pressure and temperature.

Check if the plot reaches the end of the screen. If so, clear the canvas and reset the trace to the beginning of the screen.

Java script in index.html related to decoding the six vital-sign parameters and code description are shown in Appendix B.

## 8. System Performance Test

This section concerns the performance test of our vital-sign telemedicine system. Three different performance tests were performed, namely a performance test of ECG acquisition system, a client–server communication test and a client operation test.

(i) ECG Acquisition System Performance Test

**Figure 9:**
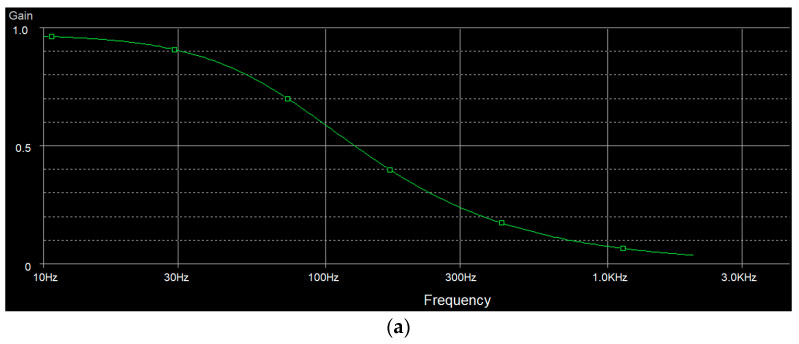

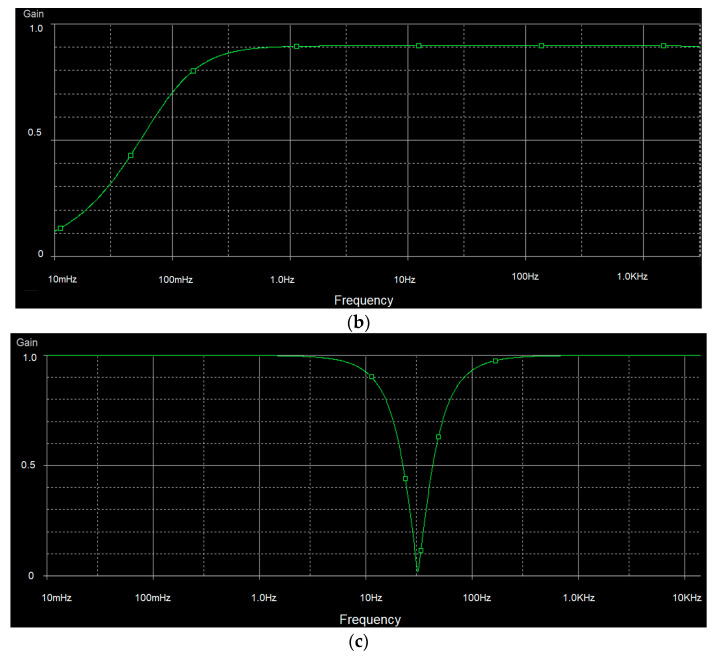
(**a**) Frequency response of 100-frequency cutoff low-pass filter, (**b**) frequency response of 0.01-frequency cutoff high-pass filer and (**c**) frequency response of 50 Hz notch filer.

ECG Acquisition system is crucial to our telemedicine system and thus requires performance tests. The first test is to measure the common mode rejection ratio (CMRR) in dB of instrumentation amplifier, which is defined as follows [22]:

CMRR = 20 log (Ad/Ac)(12)

where Ad is differential mode gain, and Ac is common mode gain. Ad is the gain when we let left-arm pin (LA) of U2 ground (GND), apply sine wave to right-arm pin (RA) of U1 and measure output of U3 (referred to Figure 1). Ac is the gain when we short RA and LA and then apply sine wave to the short pin and then measure output of U3. Our system can achieve CMRR of 75 dB. We then test the frequency response of the low-pass filter, high-pass filter and notch filter. The results are shown in Figure 9. Figure 9a shows the frequency response of the 100-frequency-cutoff low-pass filter. Figure 9b shows the frequency response of the 0.01-frequency-cutoff high-pass filter. Figure 9c shows the frequency response of the 50 Hz notch filter.

(ii) Blood Pressure Measurement Test

**Figure 10:**
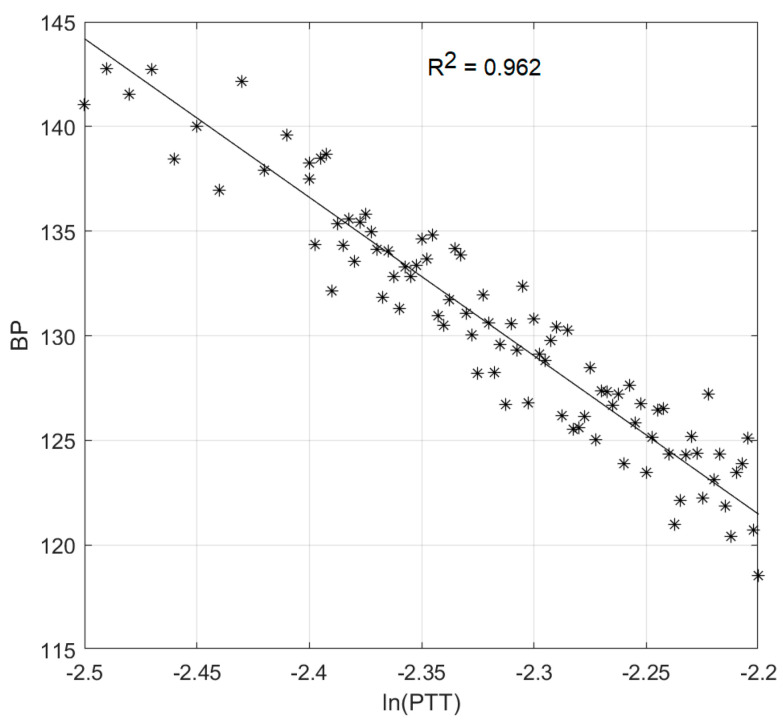
Linear-curve fitting plot of ln(PTT) versus average blood pressure.

In order to estimate the constant parameters in Equation (8), we prepared the fitting data that associates blood pressure with pulse transit time (PTT). We opted to fit only systolic blood pressure. As blood pressure seemed stable for each day, a longer period of data collection was required. We, hence, collected data for a healthy 58-year-old subject three times per day, in the period. ECG and plethysmogram are collected, using our vital-sign acquisition system. Blood pressure is collected, using a commercial automatic inflatable-cuff blood-pressure device. All digitized data are imported to MATLAB for further analysis, including peak detection of ECG and plethysmogram, PPT estimation and data fitting. The results coefficients are imported to Arduino Mega 2560 for systolic blood-pressure estimation. Figure 10 shows the linear-curve fitting plot of ln(PTT) versus average blood pressure.

(iii) Client–Server Communication Test

According to Shannon’s sampling theory [29], the sampling rate should exceed two times the maximum frequency of the sampled signal. The maximum frequency of ECG is roughly 100 Hz. In practice, for ECG acquisition, the recommended minimum sampling rate is 1000 Hz. In practice, the overall sampling rate at the client site will include the sampling rate of ADC, the baud-rate of serial communication and the internet-upload speed of Web Socket. For analog-to-digital converter (ADC), we used the MCP4725 I2C ADC module, which can provide a sampling rate of 100 kHz. For serial commination between vital-sign telemedicine acquisition system to vital-sign telemedicine web server, we set the baud rate at 115,000 bps which is equivalent to 10 kHz. The communication between vital-sign telemedicine web server and finally to vital-sign telemedicine web client exploits Web Socket, which is designed for real-time internet application. Many factors can affect the communication speed of Web Socket, such as the upload/download internet speed. We tested our system with Wi-Fi internet with a download speed of 40 Mbps, which is equivalent to sampling rate 200 kHz (40 Mbps/8 Byte per bit/25 byte per one dataset).

To evaluate the overall sampling rate, we simulated the situation by sending sine wave signal from ECG acquisition to the client. The frequency of sinewave varies from 1 to 150 Hz. At the client site, the sine wave is plotted on a canvas. Table 1 shows the cropped area of the canvas. It can be shown that our system can operate with the frequency up to 128 Hz, which is acceptable for ECG, of which the maximum frequency content is 100 Hz. At 150 Hz, an aliasing problem is clearly seen.

(iv) Client Operation Test

**Figure 11:**
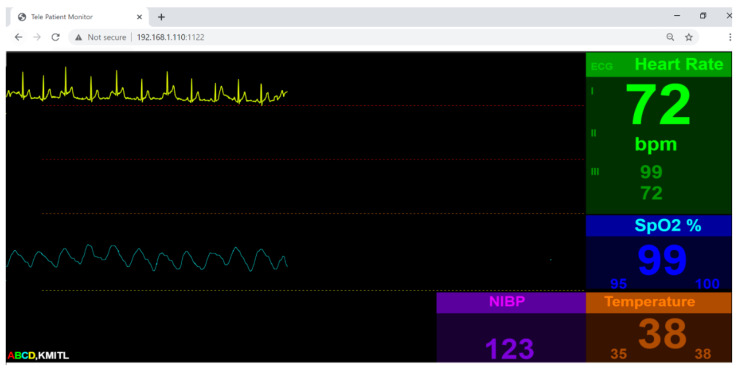
Vital-sign monitoring page.

Our vital-sign web client is designed to have three pages: (i) authentication page, (ii) database page and (iii) application page. To provide security for the user, we use Firebase authentication [30]. In order to access the vital-sign web client, the user must sign up. Upon signing up, the user’s email and password will be stored on the Firebase cloud server, to be verify for the next signing in. The database is used to register user information. The Firebase database is also used for our client database system. The data field includes name, age, address and login time. Other database fields are vital-sign information, such as average, heart rate, maximum temperature and minimum SpO2 for later investigation. The application page will be the page similar to general patient monitor. There will be two traces plotted on the html canvas and four vital sign parameters, namely temperature, heart rate, blood pressure and SpO2, displayed numerically on the web. The client page is shown in Figure 11.

**Figure 12:**
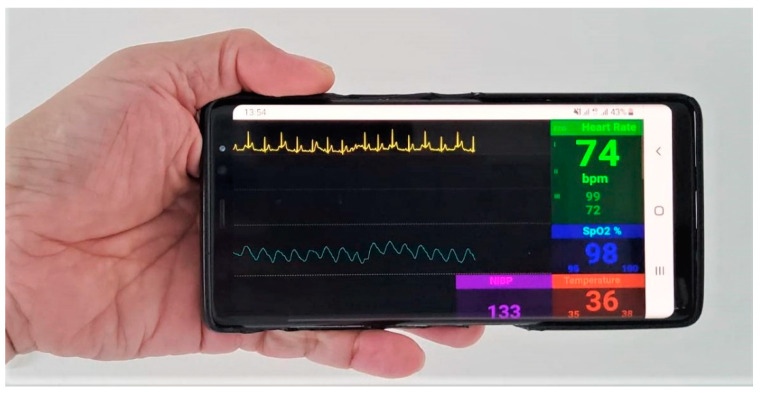
Vital-sign monitoring page on 4G Mobile Network with download speed of 20 Mbps.

Via the application of port forwarding, we also tested our vital-sign client page to access server remotely on the 4G mobile system, with a download-speed test of 20 megabits per second (Mbps), as shown in Figure 12. The download speed of over 20 Mbps will be equivalent to sampling rate of 100 kHz (20 Mbps/8 Bits per byte/25 byte per dataset). Though this is a bottle-neck sampling rate, the signal still shows no sign of delay with latency of 5 ms. However, with a download speed of less than 20 Mbps, the R wave of ECG will lose some data plot.

## 9. Discussion

The research concerned the design and construction of multi-parameter vital-sign telemonitoring exploiting Web Socket module. There are a number of issues that need to be addressed for the completeness of our research proposal:

(i) The system is operated on the LINUX platform, which can be installed on the personal computer, notebook and high-efficient microcontroller, such as Raspberry Pi. Even though the main objective of our research was to design a low-cost telemonitoring, the actual cost depends upon the device installed the LINUX platform. The notebook will offer a high performance in terms of communication speed and storage, yet the cost is higher than the embedded microcontroller. In the case of implementing on the microcontroller, the advantage will be a portable device. In such a case, a 4G Ram Raspberry Pi is recommended.

(ii) When testing the system under the Wi-Fi environment, the communication is much better than with the 4G mobile system. Wi-Fi communication can achieve communication speed of 300 Mbps, which is, on average, three to four times faster than the 4G mobile communication. The system, however, is designed to operate at the patient’s house, where Wi-Fi communication is available. In such a case, the COVID-19-infected patient will be in an isolated room, and the caretaker will be in the other room.

(iii) The system can be used to monitor many patients at the same time. In such a case, a number of vital-sign acquisition systems will be used for individual patient. All the vital-sign acquisition systems will then communicate with single vital-sign web server with unique Internet Protocol (IP) address. The client then can access to individual patient with one internet browser. To enhance performance, separated vital-sign web server can be used for each vital-sign acquisition system. In this case, the client can access each patient’s data by running a separated internet browser. The multi-patient multi-parameter vital-sign telemonitoring will be left for future works.

(iv) The accuracy of blood pressure measurement requires data collection of ECG and plethysmogram prior to the operation of the proposed system. In emergency case, the pre-computed coefficients of Equation (8) can be used. However, the correction value must be imposed by comparing the blood-pressure value with the commercial one, and later compensating to the measured blood-pressure value.

## 10. Conclusions

Under the COVID-19 pandemic, more than half of the world’s population has been forced into lockdown. Telemedicine becomes increasing significant, especially for mild-symptom COVID-19 patients or bed-ridden patients. Patient monitoring is one of the most important medical tools used to monitor vital-sign of a patient with health problem. In this research, we developed a vital-sign telemedicine system by using internet infrastructure. Our vital-sign telemedicine system consists of three main parts, namely a vital-sign telemedicine acquisition system, vital-sign telemedicine web server and vital-sign telemedicine web client. Designed to operate on a Linux operation system, the vital-sign telemedicine web server will broadcast six vital-sign information parameters sent by the vital-sign telemedicine acquisition system to vital-sign telemedicine web client, using the Web Socket package. There are three main contributions of the proposed system. Firstly, our multi-parameter vital-sign telemedicine is low in cost, making it suitable in the current situation of the COVID-19 pandemics, where the national and global economics are seriously affected by the shutdown of many economic-driven sectors. Secondly, the proposed system employed a well-known Web Socket module which is widely used for real-time internet application especially, internet of thing (IOT), for a remotely real-time patient monitors of patient infected by COVID-19, which is one the most infectious diseases since the Spanish flu. Thirdly, the system can monitor six vital signs, i.e., electrocardiogram, heart-rate, plethysmogram, percent saturation oxygen, blood pressure and body temperature, simultaneously, with no delay. The performance test of our system demonstrates that our vital-sign telemedicine system successfully operates under a Wi-Fi environment. Our system is also capable of operating under current 4G mobile technology. The system is planned for clinical test under Thailand flight-for-COVID-19 medical care.

## Figures and Tables

**Table 1:** Sampling rate evaluation.

Plot	Frequency (Hz)
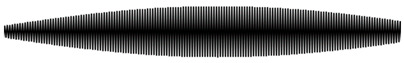	150
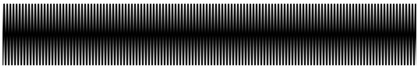	128
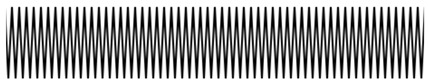	64
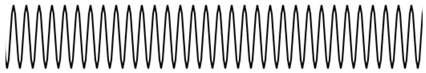	32
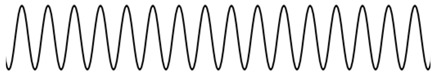	16
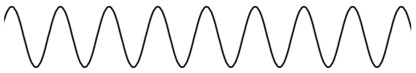	8
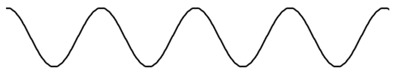	4
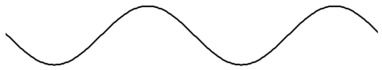	2
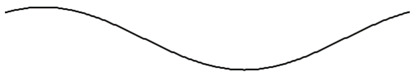	1

## Data Availability

Data available on request due to restrictions.

## Appendix A. Node.js Server Code

**Algorithms A1:** Node.js Server
1 var SerialPort = require(“serialport”);//include the Serial library 2 const fs = require(‘fs’);//include the file system library 3 //Start Serial communication 4 var myPort = new SerialPort(“com20”, { 5 baudRate: 115200, 6 dataBits: 8, 7 parity: ‘none’, 8 stopBits: 1, 9 flowControl: false 10 }) 11 const https = require(‘https’); 12 const http = require(‘http’); 13 var WebSocket = require(‘ws’); //include the webSocket library 14 //configure the webSocket server: 15 var SERVER_PORT = 1122; //port number for the webSocket server 16 var connections = new Array; //list of connections to the server 17 //Launch client index.html when connected 18 const server = http.createServer(function(req, res) { 19 fs.readFile(__dirname + ‘/public/index.html’, 20 function (err, data) { 21 if (err) { 22 res.writeHead(500); 23 return res.end(‘Error loading index.html’);24 } 25 res.writeHead(200); 26 res.end(data); 27 }); 28 }); 29 const wss = new WebSocket.Server({server}); 30 var arr = new Array(100); 31 var Readline = SerialPort.parsers.Readline;//make instance of Readline parser 32 var parser = new Readline(); 33 //make a new parser to read ASCII lines 34 myPort.pipe(parser); 35 //pipe the serial stream to the parser 36 //these are the definitions for the serial events: 37 myPort.on(‘readable’, function () { 38 arr = myPort.read(120); 39 if(arr! = null) 40 broadcast(arr); 41 console.log(arr); 42 }); 43 } 44 //------------------------ webSocket Server event functions45 wss.on(‘connection’, handleConnection); 46 function handleConnection(client) { 47 console.log(“New Connection”); //you have a new client 48 connections.push(client); //add this client to the connections array 49 client.on(‘message’, sendToSerial); //when a client sends a message, 50 client.on(‘close’, function() { //when a client closes its connection 51 console.log(“connection closed”); //print it out 52 var position = connections.indexOf(client);//get the client’s position in array 53 connections.splice(position, 1); //and delete it from the array 54 }); 55 } 56 //This function broadcasts messages to all webSocket clients57 function broadcast(data) { 58 for (c in connections) { //iterate over the array of connections 59 connections[c].send(JSON.stringify(data));//send the data to each connection 60 } 61 } 62 //this is called when new data comes into the serial port: 63 function readSerialData(data) { 64 if (connections.length > 0) { 65 broadcast(data); 66 } 67 }

## Appendix B. Java Script in Index.Html Related to Decoding the Six Vital-Sign Parameters

**Algorithms A2:** Client Java Script
1 Var obj = JSON.parse(event.data); 2 3 for (i = 0;i < 25;i++){ //loop thorough all data 4 if (obj.data[i] == 0x77 && obj.data[i + 1] = =0xbb) {//search for header 5 pkgID = obj.data[i + 3]; //Get Package ID 6 7 switch (pkgID){ //Decode 6 parameters 8 case (1): 9 ecg= obj.data[i + 4]; 10 break; 11 case (2): 12 Pleth= obj.data[i + 4]; 13 break; 14 case (3): 15 Heartrate= obj.data[i + 4]; 16 break; 17 case (4): 18 SPO2 = obj.data[i + 4]; 19 break; 20 case (5): 21 bodyTemp = obj.data[i + 4]; 22 break; 23 case (6): 24 bloodPressure = obj.data[i + 4]; 25 break; 26 27 } //Switch 28 }//for
